# Acute generalized exanthematous pustulosis with features mimicking toxic epidermal necrolysis secondary to amiodarone^☆^^☆☆^

**DOI:** 10.1016/j.abd.2019.11.010

**Published:** 2020-05-11

**Authors:** Cheryl Distel, María Luz Bollea Garlatti, Ana Clara Torre, Julia Riganti

**Affiliations:** Department of Dermatology, Hospital Italiano de Buenos Aires, Buenos Aires, Argentina

Dear Editor,

Acute generalized exanthematous pustulosis (AGEP) is an infrequent cutaneous drug eruption, with a short latency of 24–48 h between the exposure and the onset of lesions.^1^, ^2^ The symptoms consist of fever and small, sterile, non-follicular pustules on a background of erythema.^1^, ^2^ Mucous membrane and internal organ involvement are unusual.^1^, ^2^ The most common laboratory abnormality is leukocytosis and neutrophilia > 7000/mL.^2^ A score, developed by the EuroSCAR group, that takes into account clinical and histopathological criteria is useful for diagnosis.^1^, ^2^ AGEP is usually a self-limited disease, which typically resolves with cutaneous desquamation in less than 15 days after suspending the causative drug, and it has an excellent prognosis.^2^ However, although infrequent, patients can develop purpuric, targetoid, and bullous lesions, areas of denuded skin, a positive Nikolsky sign, and mucosal and multi-organ involvements, which denotes a more serious outcome.^3^ The present report describes a patient with AGEP induced by an atypical drug, who presented with this serious clinical picture.

A 69-year-old female patient, with a history of supraventricular extrasystoles, presented with fever, malaise, and small, non-follicular pustules on a background of erythema in the axillae and groin. Twenty-four hours earlier she had switched her antiarrhythmic treatment from bisoprolol to amiodarone. Upon admission, she was dyspneic and presented tachycardia, tachypnea, and suboptimal oxygen saturation. Her mucous membranes were not involved and the Nikolsky sign was negative. Her laboratory studies revealed leukocytosis (26,689 cell/mm^3^) with neutrophilia (88.25%). Blood cultures showed no growth and the chest X-ray did not reveal any abnormalities. AGEP was suspected, amiodarone was suspended, skin biopsies were obtained, and oral meprednisone 0.5 mg/kg/day was started because of her pulmonary symptoms. Histopathology revealed subcorneal pustules with no necrotic keratinocytes (Fig. 1). The EuroSCAR score was 11, compatible with definite AGEP. In spite of the initial treatment, 24 h later the patient's lesions evolved and extended. She experienced diarrhea and developed purpuric and targetoid lesions in the thighs and the gluteal area (Fig. 2); and bullous lesions that led to small erosions on her flanks (Fig. 3). Nikolsky sign was again negative. Taking into account this torpid progression, it was hypothesized that intestinal absorption of corticosteroid could not have been sufficient, the prolonged half-life of amiodarone was playing a role, and the patient could have been undergoing a different drug reaction such as toxic epidermal necrolysis (TEN) or that she could have been suffering from an overlapping of two adverse drug reactions. At this point new skin biopsies were obtained. The histopathology was again compatible with AGEP. Meprednisone dose was raised to 1 mg/kg/day and was administered intravenously. Finally, the skin lesions and systemic symptoms resolved with skin desquamation 11 days after the onset. However, after five months from the onset of symptoms, she continued to developed new recurrences every time corticosteroid was intended to be suspended.

**Figure 1:**
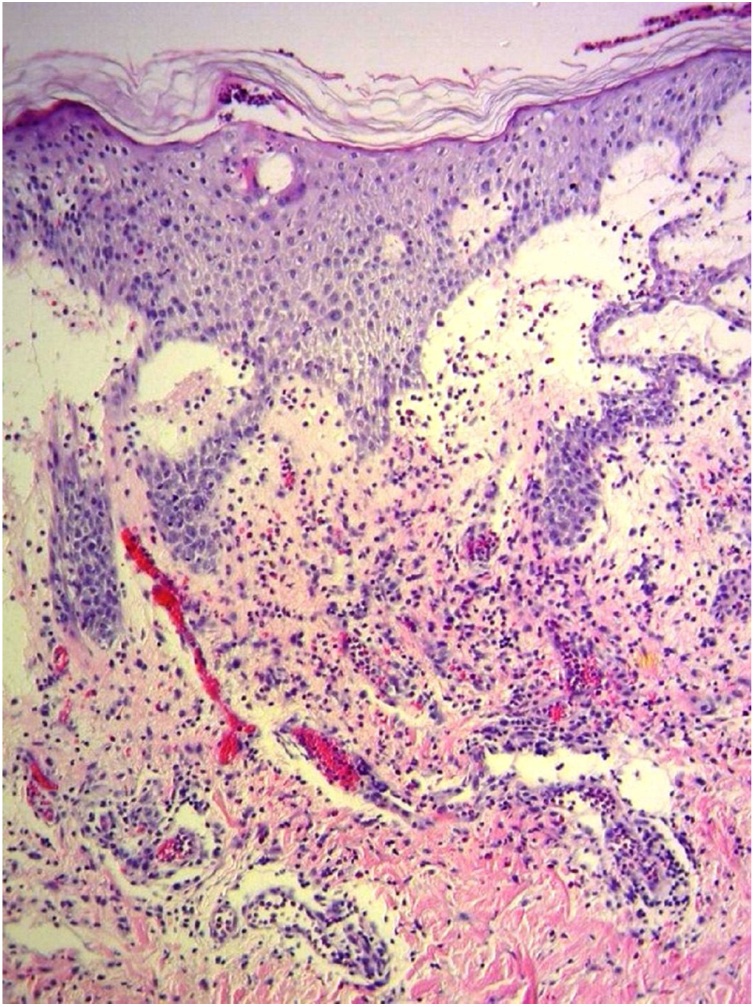
Subcorneal pustules and spongiosis (Hematoxylin & eosin, x100).

**Figure 2:**
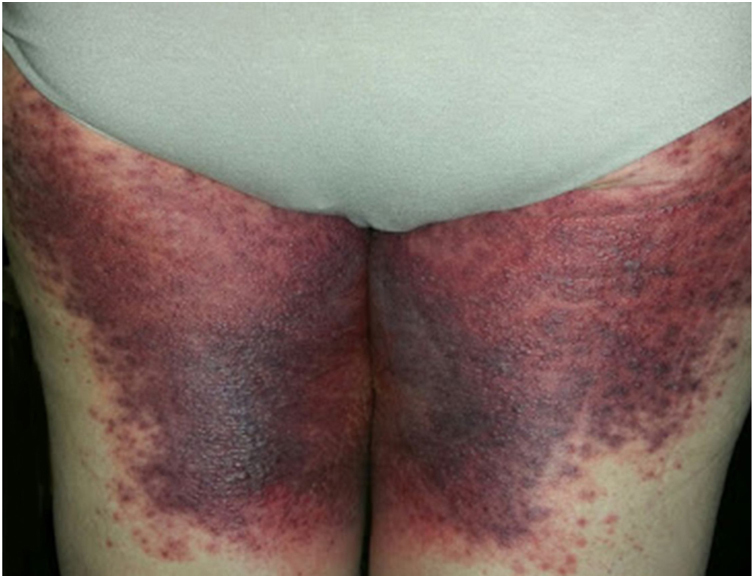
Purpuric and targetoid lesions in the thighs and the gluteal area.

**Figure 3:**
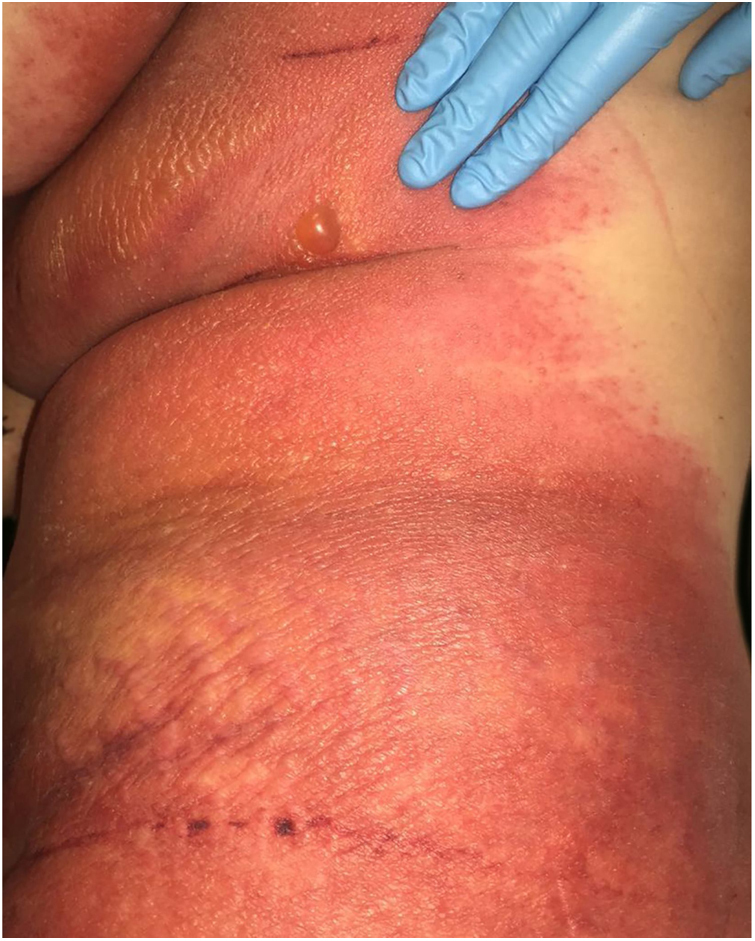
Bullous lesions that led to small erosions on flanks.

The relationship between the beginning of the new antiarrhythmic and the development of systemic and cutaneous symptoms, together with the clinical and histopathological findings, resulted in the diagnosis of AGEP induced by amiodarone. After extended research of the English and Spanish literature, to the best of the authors’ knowledge, this is the first report of AGEP triggered by this medication. Amiodarone is a fat soluble drug with a prolonged half-life of 15–142 days (mean of 58) even after the administration of a single dose.^4^ This could explain why the patient kept presenting new lesions after the discontinuation of the drug even though the medication had been suspended.

This case exhibited unusual features for classical AGEP: targetoid and bullous lesions together with torpid evolution and internal organ involvement. A diagnosis of TEN was considered, but in the absence of necrotic keratinocytes in the biopsy, it was concluded that this was actually TEN-like AGEP presentation.^5^

Although internal organ involvement is present in less than 17–20% of patients, when it occurs, hepatic and renal failure are the most common manifestations.^1^, ^2^ It can also present with respiratory symptoms^1^, ^2^ as in the present patient, in whom after ruling out infectious causes and TEN, pulmonary involvement was attributed to AGEP. Moreover, though in classical AGEP the cessation of the causative drug is the only necessary intervention, systemic corticosteroids are mandatory when organ involvement or severe cutaneous lesions are present, as in the present case.^1^

We believe that this case supports the decision of some authors to consider AGEP a severe cutaneous drug reaction and why it is important to closely follow these patients, in order to identify critical cases and intensify treatment to reduce mortality rate.

## Financial support

None declared.

## Authors’ contributions

Cheryl Distel: Drafting and editing of the manuscript; intellectual participation in the propaedeutic and/or therapeutic conduct of the studied cases; critical review of the literature; critical review of the manuscript.

María Luz Bollea Garlatti: Approval of the final version of the manuscript; drafting and editing of the manuscript; intellectual participation in the propaedeutic and/or therapeutic conduct of the studied cases; critical review of the literature; critical review of the manuscript.

Ana Clara Torre: Approval of the final version of the manuscript; intellectual participation in the propaedeutic and/or therapeutic conduct of the studied cases; critical review of the literature; critical review of the manuscript.

Julia Riganti: Approval of the final version of the manuscript; intellectual participation in the propaedeutic and/or therapeutic conduct of the studied cases; critical review of the literature; critical review of the manuscript.

## Conflicts of interest

None declared.

## References

[1] Feldmeyer L., Heidemeyer K., Yawalkar N. (2016). Acute generalized exanthematous pustulosis: pathogenesis, genetic background, clinical variants and therapy. Int J Mol Sci.

[2] Szatkowski J., Schwartz R.A. (2015). Acute generalized exanthematous pustulosis (AGEP): a review and update. J Am Acad Dermatol.

[3] Duman H., Topal I.O., Kocaturk E., Cure K., Mansuroglu I. (2017). Acute generalized exanthematous pustulosis induced by hydroxychloroquine: a case with atypical clinical presentation. An Bras Dermatol.

[4] UpToDate [Internet]. Amiodarone: drug information. Available from: https://www.uptodate.com/contents/amiodarone-drug-information [accessed 12.11.18].

[5] Kostopoulos T.C., Krishna S.M., Brinster N.K., Ortega-Loayza A.G. (2015). Acute generalized exanthematous pustulosis: atypical presentations and outcomes. J Eur Acad Dermatol Venereol.

